# Challenges in nanofabrication for efficient optical metasurfaces

**DOI:** 10.1038/s41598-021-84666-z

**Published:** 2021-03-10

**Authors:** Adelin Patoux, Gonzague Agez, Christian Girard, Vincent Paillard, Peter R. Wiecha, Aurélie Lecestre, Franck Carcenac, Guilhem Larrieu, Arnaud Arbouet

**Affiliations:** 1 CEMES-CNRS, Université de Toulouse, CNRS, Toulouse, France; 2 LAAS-CNRS, Université de Toulouse, CNRS, Toulouse, France; 3 Airbus Defence and Space, Toulouse, France; 4 LIMMS-CNRS/IIS, Institute of Industrial Science, The University of Tokyo, Tokyo, Japan

**Keywords:** Metamaterials, Silicon photonics, Nanophotonics and plasmonics

## Abstract

Optical metasurfaces have raised immense expectations as cheaper and lighter alternatives to bulk optical components. In recent years, novel components combining multiple optical functions have been proposed pushing further the level of requirement on the manufacturing precision of these objects. In this work, we study in details the influence of the most common fabrication errors on the optical response of a metasurface and quantitatively assess the tolerance to fabrication errors based on extensive numerical simulations. We illustrate these results with the design, fabrication and characterization of a silicon nanoresonator-based metasurface that operates as a beam deflector in the near-infrared range.

## Introduction

Optical metasurfaces are planar metamaterials composed of subwavelength artificial structures that resonantly couple to the incident electromagnetic field. Engineering the morphology and/or dielectric environment of these resonators allows controlling the phase, amplitude and polarization of light along the surface and yields properties that are not found in nature such as negative refraction^1–5^. The possibility to generate arbitrary wavefronts has enabled a large number of exciting applications such as beam deflection, vortex beam, hologram generation and frequency conversion^6–8^. Metasurfaces are an attractive alternative to conventional bulk optical components which offer the possibility to integrate complex optical functions in lightweight and cheap miniaturized components^9^. They hold great promise for applications such as portable or wearable devices, automotive, aeronautical and space applications and augmented/virtual reality^10–12^.

The strong losses inherent to metallic nanoresonators are a hard limit for their use in highly efficient metasurfaces^13^. Instead, metasurfaces composed of an arrangement of dielectric nanoresonators can reach much higher transmission efficiencies due to their lower intrinsic absorption in a broad spectral range^14–16^. Dielectric resonators support Mie-type optical resonances that can be of electric or magnetic character. When they spectrally overlap, these electric and magnetic optical resonances can imprint a phaseshift on an incident electromagnetic wave that spans the entire 0-$2\pi$ range and depends sensitively on the resonator morphology and environment. Dielectric metasurfaces can therefore arbitrarily tailor the wavefront of an incident electromagnetic wave with a high transmissivity. The offered possibilities have been realized with metasurfaces implementing optical functionalities of ever increasing complexity^15^. Originally limited to passive components enabling a single function and a single operation wavelength, dielectric metasurfaces have benefited from the development of sophisticated design and nanofabrication strategies^17–20^. For instance, multifunctional metasurfaces, i.e metasurfaces implementing several optical functionalities at different wavelengths or polarizations, as well as reprogrammable active metasurfaces have been demonstrated^21–27^.

Designing and fabricating optical metasurfaces requires to carefully assess the level of precision that needs to be reached to warrant the realization of the desired optical functionality with good efficiency. The discrepancies between the predicted and measured performance of metasurfaces are generally ascribed to fabrication imperfections but the relative importance of the different factors influencing their optical response is not systematically discussed. It is the purpose of our work to address this issue. In this study, we design, fabricate and characterize a silicon nanoresonator-based metadeflector operating in the near-infrared range. This type of metadevice is indeed an ideal benchmark to optimize the nanofabrication strategies that will be later used for more complex components^28–30^. The fabrication route is designed to minimize the influence of fabrication errors on the optical properties of the metasurface for the specific process window. We investigate the origin of the remaining discrepancies between the theoretical predictions and the measured values. Our work provides guidelines for the fabrication of optical metasurfaces identifying the main bottlenecks that need to be carefully addressed for optimal performance.

## Design, nanofabrication and characterization of a silicon nanoresonator-based meta-deflector

### Design of a dielectric metadeflector

**Figure 1:**
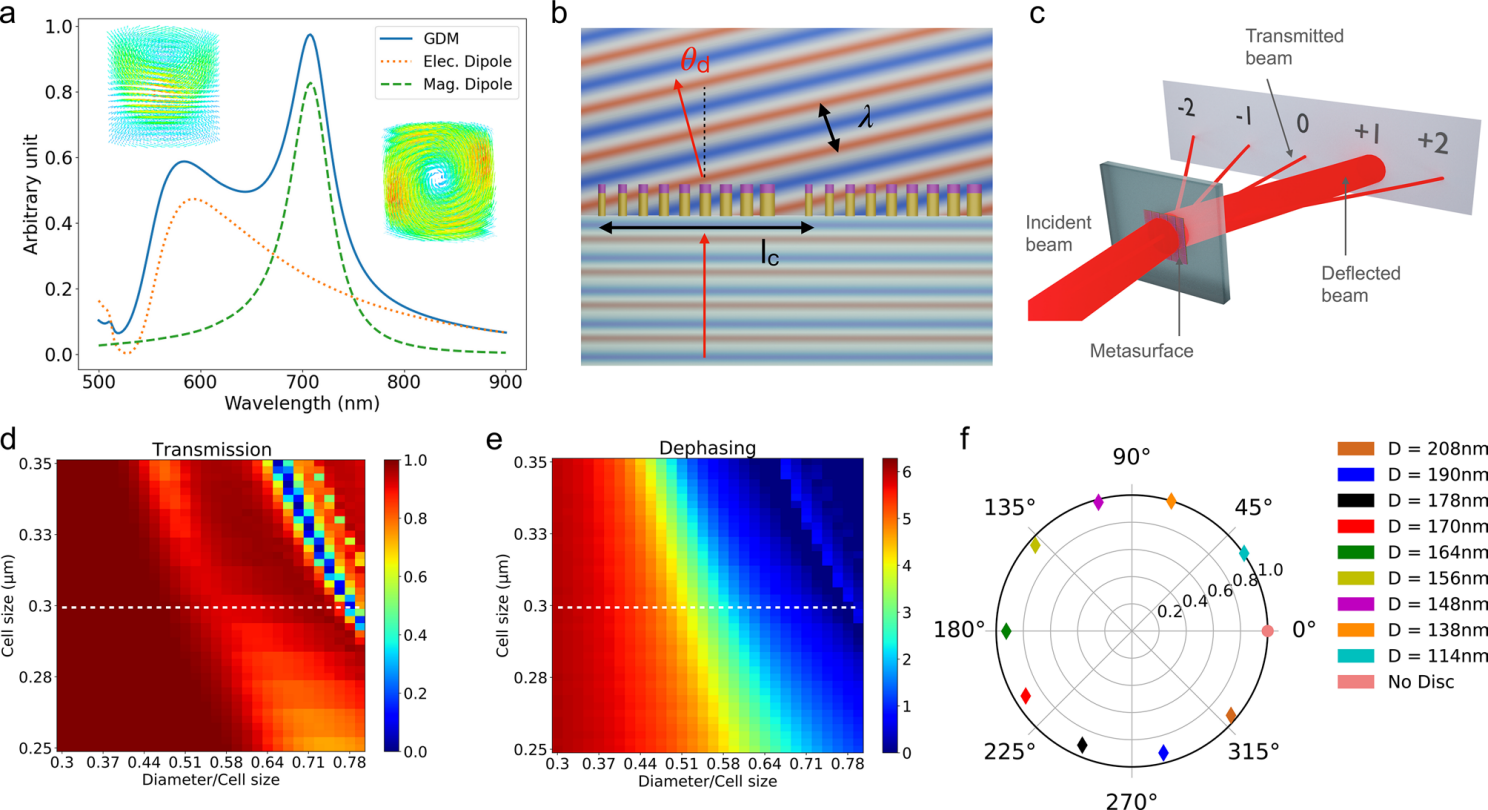
(**a**) Scattering cross-section of an isolated silicon nanocylinder ($H = 170\ \hbox {nm}$, $D =160\ \hbox {nm}$). The full line shows the total scattering cross-section whereas the orange dotted (resp. green dashed) line is the contribution from the electric (resp. magnetic) dipolar resonance only. (**b**) Deflection of a plane electromagnetic wave by a metadeflector: The phaseshift induced by the dielectric nanoresonators varies along the surface yielding a global tilt of the exit wave by an angle $\theta _d$. The metadeflector is composed of several supercells composed of $N_d$ resonators. Over the supercell length $l_c$ an incident plane wave experiences a $2\pi$ phaseshift. (**c**) A plane wave incident on a metadeflector is split into several output beams. The deflected beam carries nearly all the transmitted power. Transmission efficiency (**d**) and dephasing (**e**) of a $\lambda _{c}= 750\ \hbox {nm}$ plane wave normally incident on an infinite square array of silicon nanocylinders with constant height $H = 370\ \hbox {nm}$. The white dashed line corresponds to the lattice spacing chosen in the following. (**f**) Polar plot showing the transmission and dephasing of a $\lambda _c =750\ \hbox {nm}$ plane wave normally incident on a periodic array of identical nanocylinders with selected diameters. The height and spacing are the same for all nanocylinders ($H=370\ \hbox {nm}$ and $a =300\ \hbox {nm}$ respectively). The transmission and dephasing are respectively given by the distance to the center and angle.

Figure 1a shows the scattering cross-section of an isolated silicon dielectric nanocylinder computed using the Green Dyadic Method (pyGDM open-source numerical toolbox^31, 32^). Two optical resonances are supported. The contribution to the total scattering cross-section of the electric dipole resonance and the magnetic dipole resonance are shown separately. The vortex-like field distribution excited at the magnetic resonance is clearly visible on the 3D quiver plots of Fig. 1a. When both resonances overlap spectrally, the dephasing imprinted on an incident electromagnetic wave can span the entire 0-$2\pi$ range. In the case of high aspect-ratio dielectric nanocylinders, the $2\pi$ phase coverage involves multiple resonances^33^. Because the resonance wavelengths depend on the morphology of the nano-objects, it is possible to control this dephasing through a proper choice of the height and diameter of the nanocylinder. In the case of a 2D array of these resonators an additional control parameter adds to the parameters describing the morphology of the individual nano-objects: the distance between adjacent nanocylinder (see Supplementary Information, Fig. 1). The wavefront of an electromagnetic wave reflected or transmitted by an array of dielectric cylinders can therefore be controlled by choosing the proper shape parameters and spacing between resonators at each location (x,y) on the metasurface. This is illustrated in Fig. 1b in the case of a metadeflector composed of dielectric nanocylinders fabricated on a transparent substrate. In this case, the geometries of the nanocylinders are selected so as to imprint a phaseshift on an incident plane wave that varies linearly along a single direction *x*. As shown in Fig. 1c, the metadevice yields several output beams corresponding to different diffraction orders with nearly all the intensity contained in the deflected beam.

In the following, we design a metadeflector consisting of polycrystalline silicon nanocylinders of height *H* and diameter *D* arranged on a square lattice of spacing *a*, as sketched in Fig. 1b. As we intend to fabricate the beam deflector by top-down approach, the height *H* must be the same for all the resonators. We further choose to have the lattice spacing *a* constant over the entire metasurface to warrant fabrication and numerical simulations of reasonable complexity and duration. The metasurface is designed to operate at a wavelength $\lambda _{c} = 750\ \hbox {nm}$. To compute the optical response of the metasurface and identify an optimum set of parameters we have performed systematic Finite Difference Time Domain (FDTD) simulations (open-source package MEEP^34^) (details can be found in the Methods section). Figure 1d (resp. Fig. 1e) shows the transmission (resp. dephasing) of a normally incident $\lambda _c = 750\ \hbox {nm}$ plane wave induced by a square array of silicon nanocylinders fabricated on a glass substrate ($n_{s}=1.45$) computed as a function of the lattice spacing *a* and ratio *D*/*a*. The cylinder height is $H =370\ \hbox {nm}$. The dephasing $\Phi$ shown in Fig. 1e varies by more than $2\pi$ on the parameter space. From these maps it is possible to select $N_d$ morphologies (cylinder diameter and spacing) yielding both high transmission efficiencies and dephasings that sample regularly the 0-$2\pi$ range.

The generalized laws of reflection and refraction relate the angles of incidence, reflection and refraction to the gradient of the dephasing $\Phi$^35^. In the case of a metadeflector operating at normal incidence with air as the output medium, we have:

1 $$\begin{aligned} \sin \theta _d = \frac{\lambda _c}{2\ \pi }\ \frac{d \Phi }{dx} \end{aligned}$$

with $\theta _d$ the angle of deflection and $\lambda _c$ the vacuum wavelength of the incident electromagnetic wave. As sketched in Fig. 1b, the dephasing takes discrete values $\Phi _i$ varying from 0 to $2\pi$ over the supercell length. From equation (1), it is clear that the deflection angle is related to the supercell length $l_c$ by:

2 $$\begin{aligned} \theta _d = \arcsin \frac{\lambda _c}{l_c} \end{aligned}$$

In the following, the supercell length $l_c$ is $3\ \mu \hbox {m}$ and the wavelength $\lambda _c= 750\ \hbox {nm}$ yielding a tilt angle $\theta _d = 14.5 ^{\circ }$.

The main task when designing a metadeflector is to identify a set of parameters (height *H*, lattice spacing *a*, diameters $(D_i)_{1\le i \le N_d}$) yielding a linear phase ramp $(\Phi _i)_{1\le i \le N_d}$ and the best transmission possible. This set of parameters is not unique and several factors need to be taken into account. First, the number of different geometries $N_d$ used to sample the 0-$2\pi$ range obeys two opposite constraints. It is well known from the theory of diffractive optical elements that the maximum diffraction efficiency of a discretized sawtooth grating varies with the number of phase steps $N_d$ as $\mathrm{sinc}^2\left( 1/N_d \right)$. This sets an upper limit for the achievable diffraction efficiency of 98 % in the case of $N_d =10$ phase discretization elements. On the opposite, larger values of $N_d$ mean closer values of the parameters describing the morphology of the cylinders and therefore require a higher level of fabrication precision. For a given lattice spacing, larger nanocylinder diameters will yield smaller gaps between resonators that may lead to the formation of bridges between the nano-objects. We set $N_d = 10$ as a compromise between efficiency and compatibility with the fabrication technique. As stated previously, we have chosen to keep the lattice spacing *a* constant over the entire metasurface. We have further tried to minimize the sensitivity to fabrication imperfections by minimizing the gradients that relate the dephasing to changes in nanocylinder diameter and height. These considerations lead us to the choice of a lattice spacing $a = 300\ \hbox {nm}$ and the following cylinder diameters: 114, 138,148,156,164,170,178,190, 208 nm. At the tenth position of the supercell we decided to have no cylinder yielding a null dephasing and a transmission efficiency of 1. The computed phase shift and transmission of the selected nanocylinder array geometries are represented on a polar plot in Fig. 1f. This figure shows that the chosen configurations regularly sample the 0-$2\pi$ interval with a good transmission.

### Fabrication procedure

**Figure 2:**
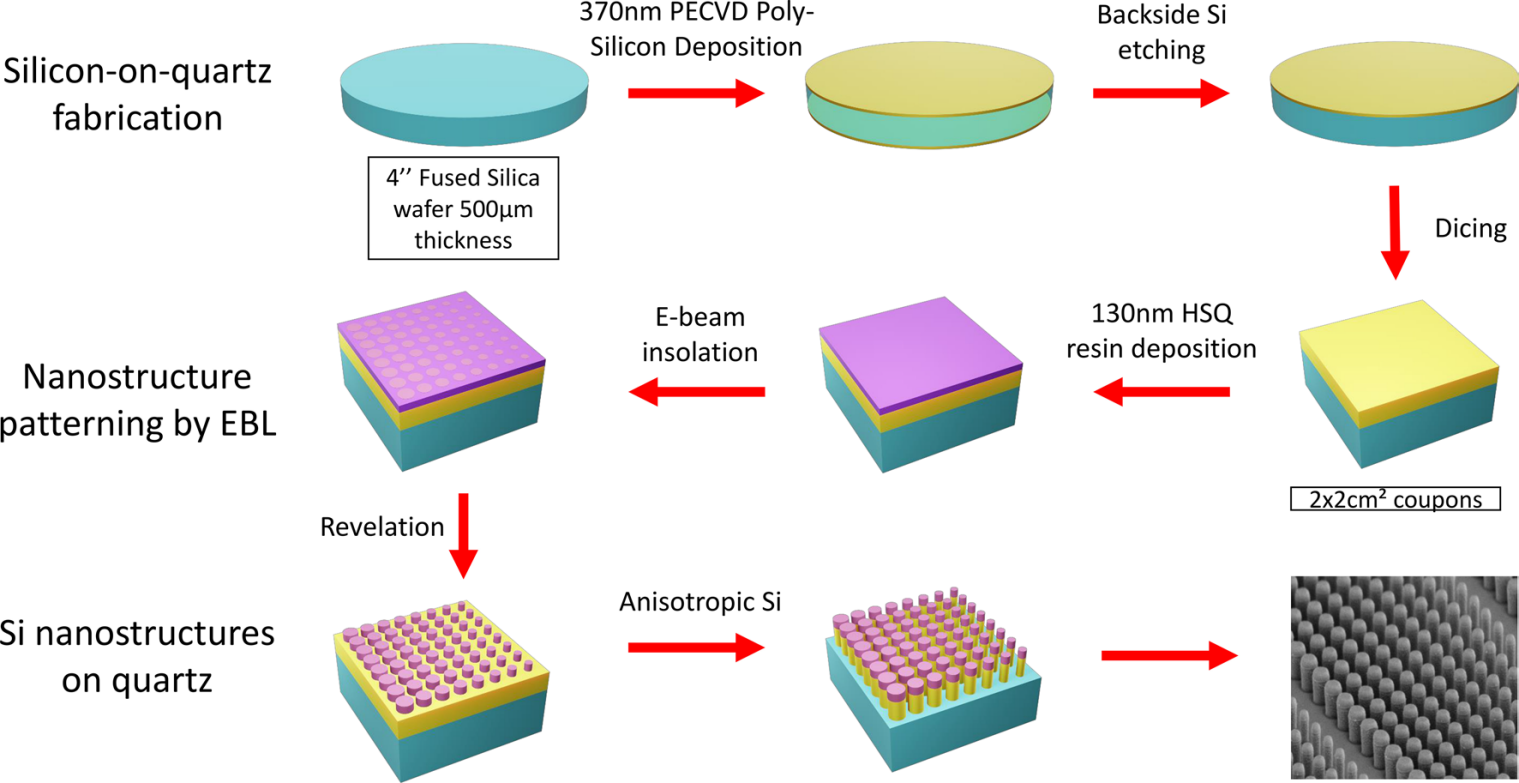
Top-down fabrication of a metadeflector composed of silicon nanocylinders on a quartz substrate.

The fabrication method is described in Fig. 2. The process starts with the engineering of a pseudo Silicon on quartz wafer. A 370 nm thick polycristalline Si layer is deposited by Low Pressure Chemical Vapor Deposition (LPCVD) on a 4 inch fused silica wafer. The thickness and the refractive index of the deposited layer are measured by ellipsometry. The Si layer on the back side is selectively etched and the 4” wafer is diced down to 2cm x 2cm chips.

The nanostructuration is performed by top-down approach on each chip. We use a negative-tone resist, namely Hydrogen-Silsesquioxane (HSQ). First, a 130 nm thick HSQ layer is deposited by spin coating, and annealed on hot plate at $80\ ^{\circ }\hbox {C}\,/\,1$ min in order to evaporate the solvent. The Electronic Beam Lithography (EBL) was carried out with a RAITH 150 writer at 30 keV energy exposure with a dose ranging from 855 to $1260\ \mu \mathrm{C}/\mathrm{cm}^{2}$ and a beam current of 120 pA. The resist development was performed in high concentrated (25%) TetraMethylAmmonium Hydroxide (TMAH) to increase the pattern contrast^36^. Finally, the wafer was rinsed in deionized water then in methanol solution before a soft dry with nitrogen flux in order to reduce the surface tension and minimize the nanopillar collapse^37^. Finally, the HSQ patterns are transferred in the Si layer by anisotropic plasma etching down to the quartz interface using a fluorine chemistry ($\hbox {SF}_{6}/\hbox {C}_{4}\hbox {F}_{8} /\hbox {O}_{2}$). The plasma etching is achieved by ICP-RIE (Alcatel-AMS4200 equipment) using fluorine gases. The metadeflector has a square footprint with an edge length of $500 \mu \hbox {m}$. A Scanning Electron Microscope (SEM) image of the metadeflector is shown in the bottom right hand corner of Fig. 2.

### Characterization of the metadeflector:

**Figure 3:**
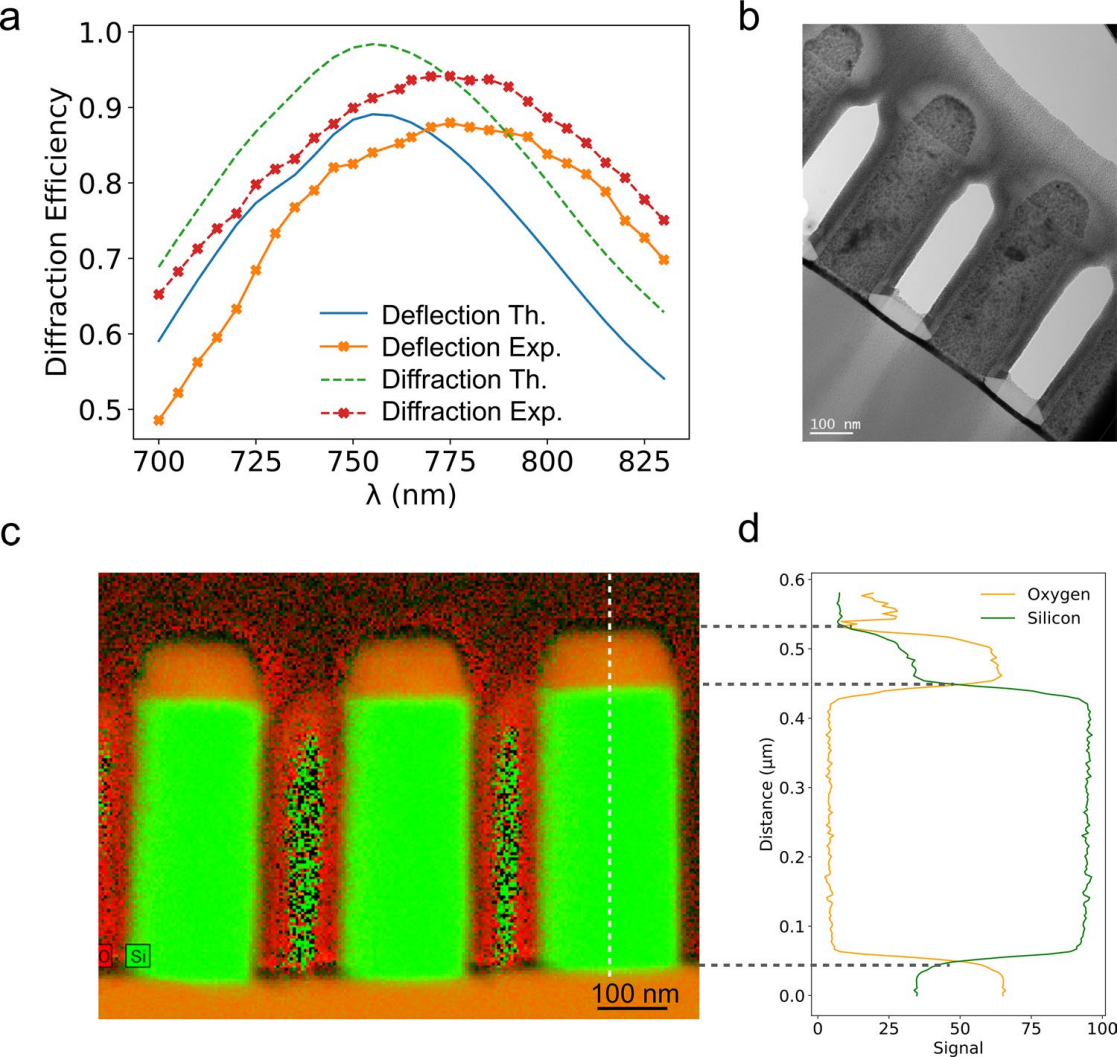
(**a**) Computed (solid and dashed line) and measured (symbols) metasurface diffraction and deflection efficiencies as a function of the wavelength of the incident plane wave. (**b**) TEM image of two silicon nanocylinders obtained by FIB cross section. (**c**) Elemental map measured by STEM-EDX showing the location of silicon and oxygen. (**d**) Elemental composition profile taken along the white dashed line visible in (**c**).

We first characterize the optical performance of the fabricated component. To do so, we use a Ti:Sa femtosecond laser tunable in the near-infrared range (680–1080 nm). A telescope is inserted in the optical path to make the laser beam smaller than the metasurface. As sketched in Fig. 1c, at the exit of the metasurface, the laser beam is deflected by an angle $\theta _{exp} = 14.5^{\circ }$ matching the expected value. Additional beams corresponding to different diffraction orders are barely visible, the corresponding intensities being much smaller than the one of the principal, deflected, beam. The transmitted power $P_{trans}$ is the sum of the power corresponding to these different beams. The diffraction efficiency is the ratio of the power $P_{d}$ of the “useful” beam, i.e exiting the metasurface at the correct angle to the transmitted power $P_{trans}$. We define the deflection efficiency as the ratio $P_{d}/P_{inc}$, $P_{inc}$ being the power incident on the metasurface. The transmission efficiency is the ratio between the incident and transmitted power $P_{trans}/P_{inc}$. We have systematically measured these quantities as a function of the wavelength of the incident beam. Figure 3a shows the computed and measured diffraction and deflection efficiencies as a function of the incident wavelength. As expected for a metasurface based on nanoresonators, the diffraction efficiency strongly depends on the incident wavelength. The computed diffraction (resp. deflection) efficiency reaches a maximum value of 98 % (resp. 89 %). The values measured in our experiments (94 % and 88 % respectively) lie below these theoretical values but are among the highest reported for silicon nanoresonator based metadeflectors operating in the near-infrared range^29^. We notice that the maximum deflection efficiency is obtained at $\lambda _{inc} = 775\ \hbox {nm}$ whereas our metasurface was originally designed for an operation wavelength of 750 nm. Similar shifts have already been observed in the literature but their origin was not addressed in details^29, 38^.

To elucidate the origin of the observed discrepancies, we have performed Transmission Electron Microscopy (TEM) experiments to precisely characterize the morphology and environment of the nanoresonators. The TEM experiments have been performed on a Philips CM20 FEG TEM at 200 keV. A cross-section lamella has been prepared by Focused Ion Beam (FIB) and micromanipulation. A bright field image of two neighbouring silicon nanocylinders is shown in Fig. 3b. We have systematically noticed that the diameters measured at the bottom of the nanocylinders are smaller than the ones measured at the top, the difference lying between 5 and 10 nm. However, the average diameter is always larger by less than 5 nm than the targeted ones confirming the excellent control during fabrication. To go further, we have performed elemental mapping using energy dispersive X-ray imaging in Scanning Transmission Electron Microscopy mode (STEM-EDX). An example of the elemental maps is displayed in Fig. 3c which shows the distribution of silicon and oxygen in the sample. These maps and the extracted profiles shown in Fig. 3d reveal the presence of an HSQ capping remaining on top of the cylinders. The latter is close to $\hbox {SiO}_{2}$ compound after modification by the electron beam, as shown in Fig. 3d. A value of 100 nm for the thickness of this remaining resist layer has been extracted from the TEM images. It highlights that our approach can be implemented on thicker Si layer (around $1\ \mu \hbox {m}$ thick) without modification of the EBL patterning condition. A few nanometer thick silicon oxyde layer is also visible on the sides of the resonators.

Both the optical characterization and TEM investigations confirm the excellent match between the fabricated metasurface and its numerical model. A close inspection of the TEM results nevertheless suggests several small differences such as (i) difference in diameter and height between experiment and theory, (ii) difference in diameter between the top and bottom of the cylinders, i.e inclination of the nanocylinder sides, (iii) capping by remaining HSQ, (iv) presence of a silicon dioxyde layer on the sides. In the following, we perform systematic numerical simulations to selectively investigate the influence of these fabrication imperfections on the optical performance of the device.

## Influence of fabrication imperfections on the optical response of metasurfaces

As demonstrated by the TEM investigations described above, several factors can alter the performance of an optical metasurface with respect to the predictions of the numerical simulations. First, the morphological parameters of the nanoresonators (diameter, height) not only differ between experiment and theory but also vary between supposedly identical nano-objects. Second, the shape itself can be different from the expected nanocylinder with in-plane (elliptical profile instead of circular) or out-of-plane (lateral face not orthogonal to substrate) imperfections. Lastly, the dielectric properties of the resonator itself, the substrate or the environment of the resonators (remaining HSQ used as a mask for etching and not removed after fabrication process) might not be perfectly accounted for in the simulations. In the following, we address the influence of each of these fabrication imperfections through systematic numerical simulations using the FDTD technique. As before, we consider a square array of silicon nanocylinders fabricated on a quartz substrate but we selectively modify either the cylinder morphology, environment or the dielectric properties. The simulation parameters (illumination, simulation, volume) are unchanged with respect to the original simulations. In this part, we assume that all nanocylinders are altered in an identical fashion. We address the influence of a statistical distribution of shape parameters among the nano-objects in the last part of this study.

### Influence of the nanoresonator morphology

**Figure 4:**
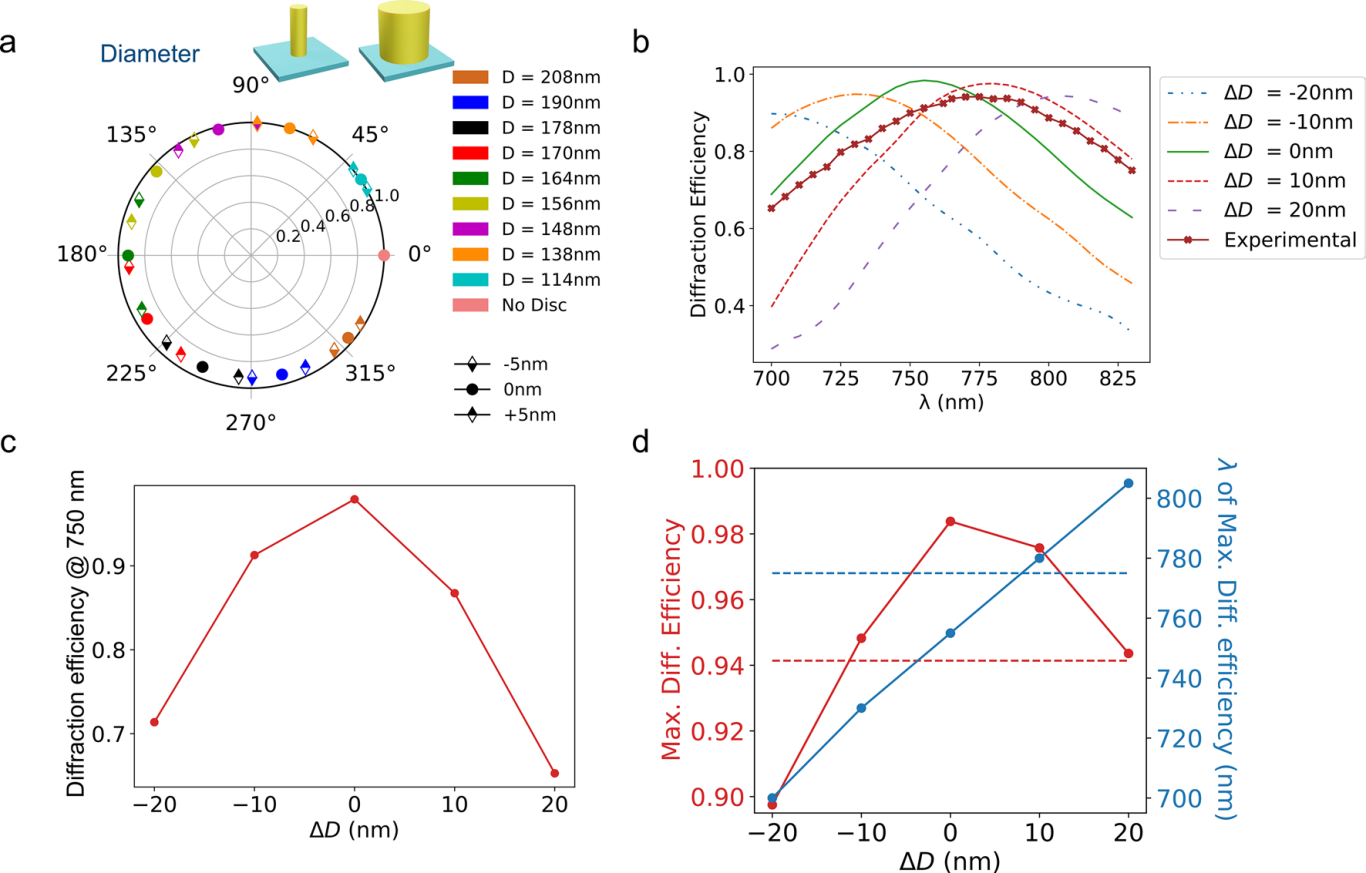
(**a**) Transmission and dephasing induced by an array of silicon nanocylinders on a normally incident $\lambda = 750\ \hbox {nm}$ plane wave as a function of a change in diameter for the ten selected geometries. (**b**) Diffraction efficiency of a metadeflector in which the Si nanocylinders have their diameter modified from the original value by the same amount as a function of incident wavelength. (**c**) Diffraction efficiency at the design wavelength $\lambda _c = 750\ \hbox {nm}$ as a function of the difference in diameter. (**d**) Maximum diffraction efficiency and wavelength of max. diffraction efficiency as a function of difference in diameter. The red (resp. blue) dotted lines show the measured values of maximum diffraction efficiency and corresponding wavelength.

We first investigate the influence of a change in nanocylinder diameter. Figure 4a shows the transmission efficiency and dephasing of a square array of identical silicon nanocylinders having a diameter slightly modified with respect to its original value for the ten geometries selected above. It is clear from Figs. 4a and b that the cylinder diameter has a strong influence on the induced dephasing: differences in diameter as small as 10 nm yield differences in the induced dephasing close to $\pi /4$. The phase ramp associated with the selected geometries becomes completely mixed up. Figure 4b shows the diffraction efficiency of a metadeflector in which *all* nanocylinders have been fabricated with a systematic difference in diameter with respect to the nominal values. The wavelength of maximum diffraction efficiency typically shifts by 25 nm for differences in diameter as small as 10 nm. Figure 4c shows that at the design wavelength of 750 nm, the diffraction efficiency decreases by 10–15 % if the diameter of the nanocylinders is changed by 10 nm and drops by up to 30 % if the diameter change reaches 20 nm. Figures 4c–d show that the fabrication of high performance optical metasurfaces requires a level of precision better than 10 nm.

**Figure 5:**
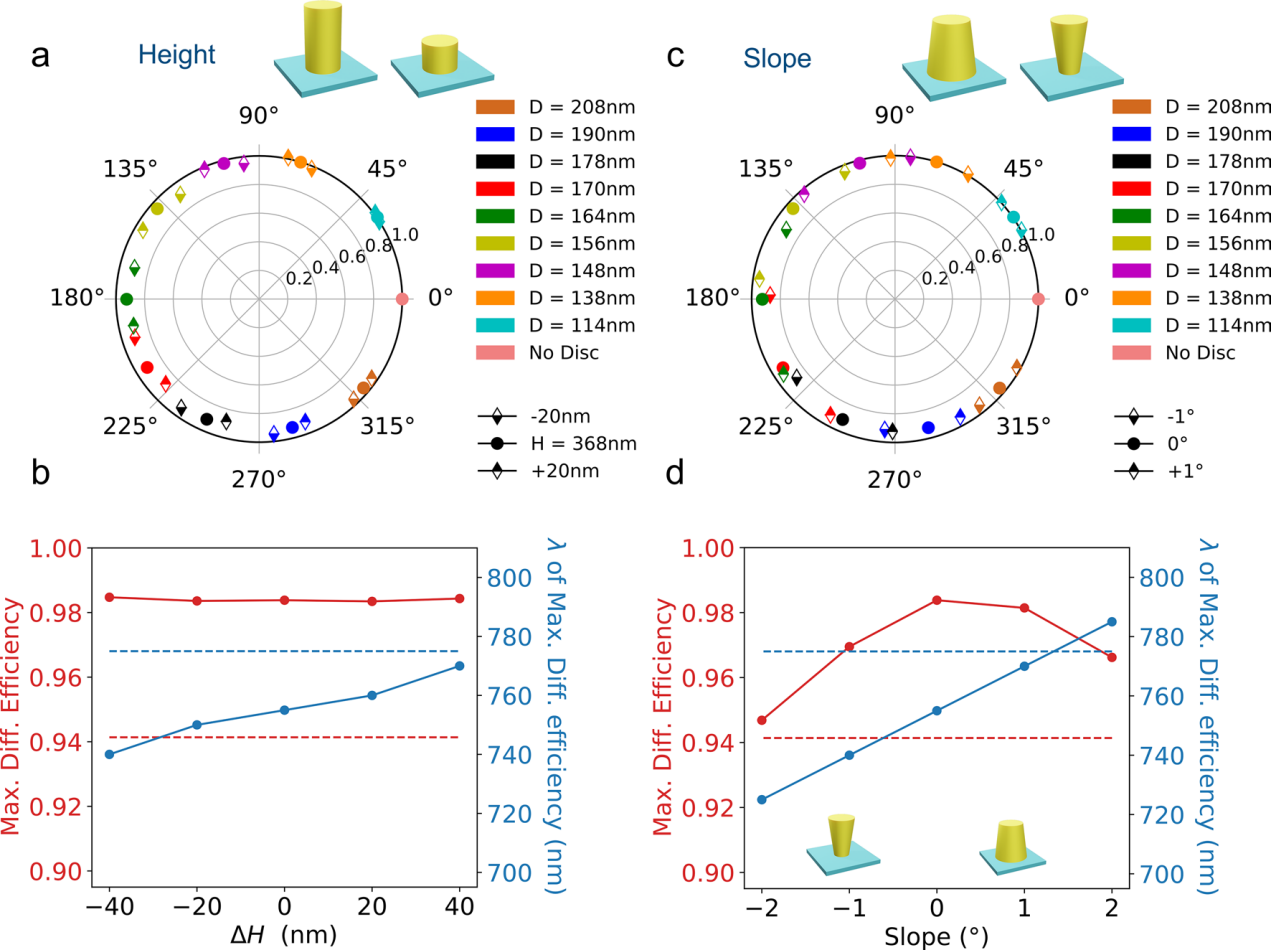
(**a**) Transmission and dephasing induced by an array of silicon nanocylinders on a normally incident $\lambda = 750\ \hbox {nm}$ plane wave as a function of a change in height for the ten selected geometries. (**b**) Diffraction efficiency and wavelength of max. diffraction efficiency as a function of difference in height. (**c**) Transmission and dephasing induced by an array of silicon nanocylinders on a normally incident $\lambda = 750\ \hbox {nm}$ plane wave as a function of a change in lateral slope for the ten selected geometries. (**d**) Diffraction efficiency and wavelength of max. diffraction efficiency as a function of the slope of the cylinder side. The red (resp. blue) dotted lines show the measured values of maximum diffraction efficiency and corresponding wavelength.

We then address the influence of a change in height on the optical response of the metasurface. All other parameters (lattice spacing, diameter, dielectric properties) are unchanged. The results are shown in Fig. 5-a in polar coordinates for the selected geometries. This figure shows that neither the transmission nor dephasing induced by the array of nanoresonators changes drastically upon modification of the height. The maximum diffraction efficiency and associated wavelength are shown in Fig. 5b. The wavelength of maximum diffraction efficiency gradually shifts towards longer wavelengths with increasing height. We notice that the value for the wavelength of maximum diffraction efficiency measured in our experiments is not reached even for a height difference of 40 nm. Figure 5b also shows that the maximum diffraction efficiency is barely affected by a change in height.

The influence of a slope of the nanocylinder sides is then investigated. Figure 5c shows the influence of a $\pm 1^{\circ }$ tilt on the transmission and dephasing of the ten selected geometries. It shows that the transmission efficiency of a silicon resonator array is weakly affected by a tilt of its sides. On the contrary, a strong influence is visible on the dephasings. We note that the experimental tilt extracted from the TEM images is of the order of $1^{\circ }$: a 10 nm change in diameter for a cylinder of 370 nm in height is $0.8^{\circ }$. As shown in Fig. 5d, the diffraction efficiency is maximum at the design wavelength $\lambda _c = 750\ \hbox {nm}$ and the wavelength of maximum diffraction efficiency shifts by 15 nm per degree of lateral slope. In the TEM observations, we have noticed that the average diameter of the cylinders are larger than the nominal values and that the bottom diameters are smaller than the top ones corresponding to the negative lateral slopes displayed in Fig. 5d. In this situation, our simulations show that the blueshift associated with negative slopes can partially mitigate the redshift associated to larger average diameters.

### Influence of the dielectric properties

**Figure 6:**
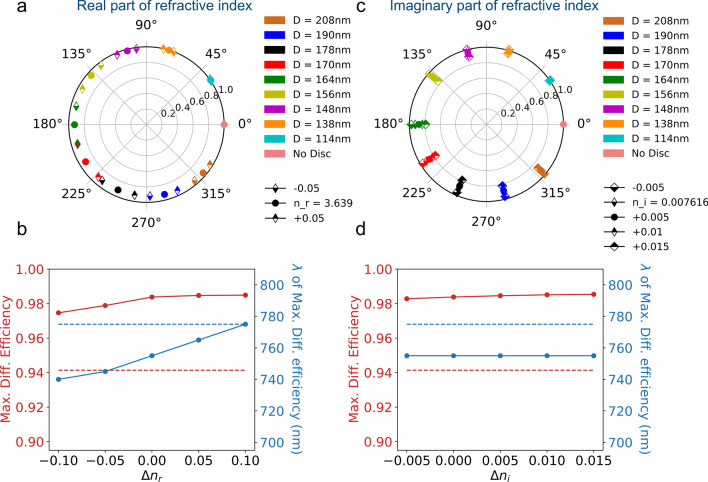
(**a**) Transmission and dephasing induced by an array of silicon nanocylinders on a normally incident $\lambda = 750\ \hbox {nm}$ plane wave as a function of the real part of the refractive index for the ten selected geometries. (**b**) Diffraction efficiency and wavelength of max. diffraction efficiency as a function of the real part of the refractive index. (**c**) Transmission and dephasing induced by an array of silicon nanocylinders on a normally incident $\lambda = 750\ \hbox {nm}$ plane wave as a function of the imaginary part of the refractive index for the ten selected geometries. (**d**) Diffraction efficiency and wavelength of max. diffraction efficiency as a function of the imaginary part of the refractive index. The red (resp. blue) dotted lines show the measured values of maximum diffraction efficiency and corresponding wavelength.

Beside differences in resonator geometry, the TEM experiments have revealed that the silicon nanocylinders have a 100nm remaining HSQ layer on the top and a few nanometers thick silicon dioxyde on their sides. Furthermore, the refractive index values considered in our simulations may not exactly match the one of the fabricated nanostructure. Therefore, we now look at the influence of errors in the dielectric constants of both the material constituent of the nanoresonators and its environment.

Figure 6a shows the influence of the real part of the refractive index of the nanocylinder material on the transmission efficiency and dephasing for the ten selected geometries. A modification of $n_r$ by approximately $1 \%$ has a weak influence on the transmission efficiency and a limited one on the induced dephasing. An increase of $n_r$ induces a redshift of the wavelength of maximum diffraction efficiency visible on Fig. 6b. Figure 6c shows the influence of the imaginary part of the refractive index of the nanocylinders. Changing $n_i$ has clearly no impact on the induced dephasing. On the opposite, increasing $n_i$ clearly decreases the transmission efficiency of the nanocylinder array. The modification of $n_i$ has no influence on the wavelength of maximum diffraction of the metadeflector and a very weak one on the diffraction efficiency as illustrated in Fig. 6d. The latter observation is consistent with the fact that the change in $n_i$ does not modify the induced dephasings. However, the poorer overall transmission efficiency associated to larger values of $n_i$ would have a visible impact on the deflection efficiency, i.e the ratio of the power in the deflected beam to the power incident on the metasurface.

**Figure 7:**
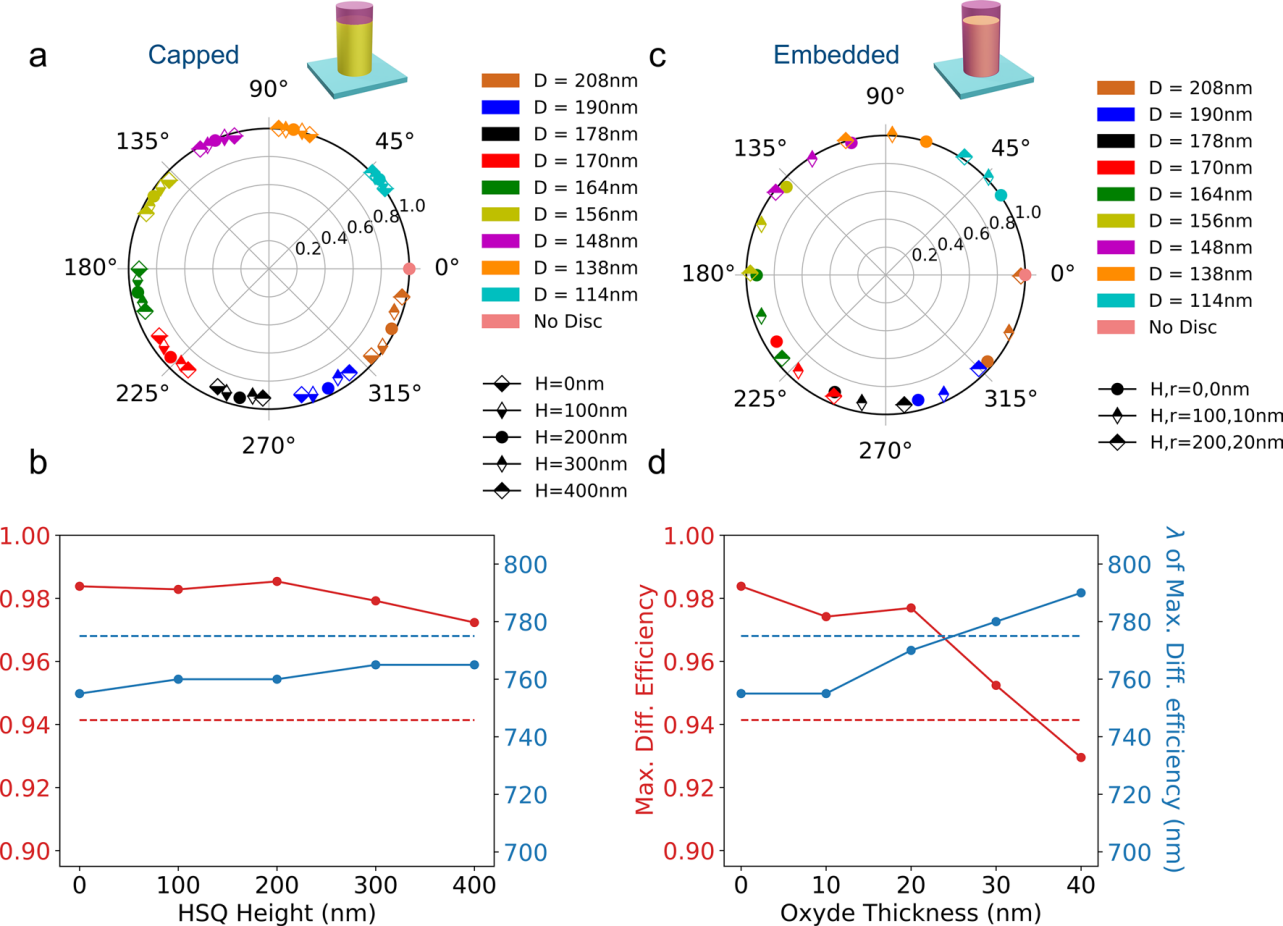
(**a**) Transmission and dephasing induced by an array of silicon nanocylinders on a normally incident $\lambda = 750\ \hbox {nm}$ plane wave as a function of the thickness of a residual resist layer for the ten selected geometries. (**b**) Diffraction efficiency and wavelength of max. diffraction efficiency as a function of the thickness of a remaining resist layer. (**c**) Transmission and dephasing induced by an array of silicon nanocylinders on a normally incident $\lambda = 750\ \hbox {nm}$ plane wave as a function of the thickness of a remaining resist layer and lateral oxyde layer for the ten selected geometries. (**d**) Diffraction efficiency and wavelength of max. diffraction efficiency as a function of the thickness of a remaining resist layer and lateral oxyde layer . The red (resp. blue) dotted lines show the measured values of maximum diffraction efficiency and corresponding wavelength. In Figures c and d, the capping thickness is taken ten times larger than the thickness of the lateral oxyde layer.

We now study the influence of the dielectric environment of the nanocylinders. First, we have performed numerical simulations taking into account an additional resist capping on top of the silicon nanocylinders. The results are given in Fig. 7a–b as a function of the thickness of the capping. The presence of this remaining layer has a negligible effect on the transmission and dephasing of the Si nanocylinder array for the ten selected geometries even for the large thickness values considered. A 400 nm thick resist layer induces a redshift of the maximum diffraction wavelength by only 10 nm and a decrease of the maximum diffraction efficiency by 1 $\%$. We now consider an additional oxyde layer on the side of the cylinder. The situation is different in this case (Fig. 7c) and even a 10 nm thick oxyde layer already has a significant influence on the predicted dephasings.

Figure 7d shows that this additional oxyde layer impacts both the maximum diffraction efficiency and corresponding wavelength. This higher sensitivity to a modification of the dielectric environment on the lateral face of the cylinder compared to the top is related to the distribution of the electric field excited in the nano-object.

The blue dotted lines in Figs. 4–7 display the wavelength of maximum diffraction efficiency measured in our experiments. Our simulations show that, beside the cylinder diameter, the slope of their lateral face or the presence of an oxyde layer on the side are potential contributors to the observed redshift of the measured wavelength of maximum diffraction efficiency. The TEM observations reported above point toward these small deviations. The simulations also suggest that a change in height or the presence of a remaining mask layer on top of the nanocylinders have a surprisingly weak influence on the final optical performance. Finally, we have performed FDTD simulations taking into account the combined influence of these different factors based on the dimensions extracted from the electron microscopy experiments. The results given in Supplementary Information confirm that the observed spectral shift of the wavelength of maximum diffraction is well accounted for by taking into account the actual geometry of the fabricated cylinders (SI, Fig. 2).

### Impact of statistical fluctuations in the nano-resonator dimensions

We have considered so far arrays of identical silicon nanocylinders and selectively looked at the influence of *systematic* modifications of their shape or dielectric properties on their optical response. Beside these effects, random variations of the individual nano-objects are expected to impact the optical response of a metasurface.

**Figure 8:**
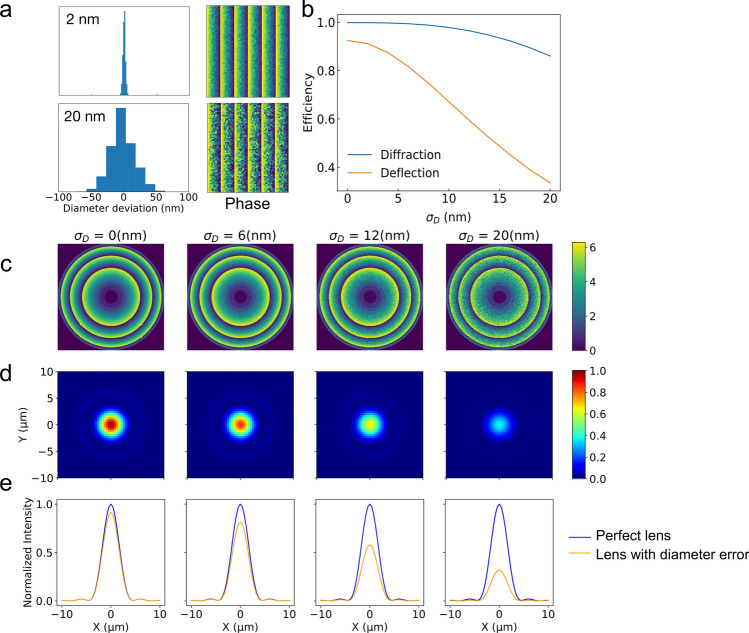
(**a**) Statistical distribution of diameter change (left) and corresponding map of the dephasing induced by the metadeflector (right) for two different values of $\sigma _D$. (**b**) Deflection and diffraction efficiency of a $100 \times 100 \, \mu \mathrm{m}^2$ metadeflector as a function of the standard deviation on the nanocylinder diameters. The metadeflector nominal supercell is the same as before. (**c**) Map of the dephasing induced by a metalens (diameter $100 \, \mu \mathrm{m}$, focal distance $500 \, \mu \mathrm{m}$) for different values of $\sigma _D$. (**d**) Corresponding intensity distribution in the focal plane. (e) Brown line: Transverse intensity profile in the focal plane of the metalens. Blue line: case of a perfect lens.

Here, we investigate the influence of these inhomogeneities on the performance of the metadeflector. The nanocylinders are as before located on the nodes of a square array of pitch *a*. We assume that these nanocylinders behave as a collection of diffracting elements placed on a square grid at locations $(x_i , y_j)$ with $x_i = i a$ and $y_j = j a$ and compute the far-field response of the device in the framework of the Fresnel-Kirchhoff theory of diffraction. We assume that the metadeflector is, as before, composed of supercells of nine cylinders of diameter $D_k$ plus an empty space. We now allow for random variations of the cylinder diameter so that $D_{ij}= D_{0,ij} +\delta _{ij}$ in which $D_{0,ij}$ is the nominal value of the cylinder diameter extracted from the FDTD simulations presented above. The fluctuations of the nanocylinder diameters $\delta _{ij}$ are described by a gaussian distribution, the standard deviation of which accounts for the precision of the fabrication process. To reproduce the influence of fluctuations of the diameter of the cylinders around their nominal values, we have interpolated the dependence of the transmission efficiency and dephasing on the diameter from the systematic FDTD simulations presented above. From the dependencies of transmission and dephasing with the diameter of the nanocylinders, it is possible to calculate for a given distribution of nanocylinder diameters $D_{ij}$ on the metasurface the transmission and dephasing induced at position $(x_i ,y_j )$. Fig. 8a shows the statistical distribution of diameter changes $\delta _{ij}$ for two different values of the standard deviation $\sigma _D$. The corresponding phase maps are given on the right and show the degradation of the deflector phase ramp for increasing values of $\sigma _D$. From the distribution of transmission efficiency and dephasing on the surface of the metadevice we have calculated the diffraction efficiency and deflection efficiency for different values of the standard deviation on the nanocylinder diameters. The results presented in Fig. 8b are computed for a $100 \times 100 \, \mu \mathrm{m}^2$ metasurface having a pitch $a = 300\ \hbox {nm}$. They show that fluctuations on the nanocylinder diameters deteriorate both diffraction and deflection efficiencies. A precision on the nanocylinder diameter better than 5 nm is mandatory to keep the deflection efficiency above 80 %. A standard deviation on the diameter distribution of 10 nm degrades the deflection efficiency by 20 %. In the Supplementary Information, we have studied the influence of random errors on the position of the nanocylinders and stitching errors of the writing fields during the electron beam lithography process (SI, Fig. 3). Our results show that these effects do not contribute significantly to the degradation of the optical performance in our experimental conditions.

Finally, we have addressed the influence of fabrication precision in the case of a metalens composed of silicon nanocylinders on a quartz substrate. The metalens has a diameter of $100 \mu \hbox {m}$ and a focal distance of $500 \mu \hbox {m}$ and is designed to operate at $\lambda _c = 750\ \hbox {nm}$. We have used the transmission and dephasing maps shown in Fig. 1d–e to select the geometries of the nanocylinders composing the metalens. We have studied the focusing properties of the metalens as a function of the errors on the nanocylinder diameter using the same approach as the one used for the metadeflector. We assume that the diameters are distributed around their theoretical values according to a gaussian distribution of standard deviation $\sigma _D$. Figure 8c shows the dephasing imprinted on a $\lambda _c = 750\ \hbox {nm}$ incident plane wave by the metalens for different values of $\sigma _D$. Figure 8d shows the corresponding distribution of intensity in the focal plane of the metalens and Fig. 8e shows the intensity profile together with the one of a perfect lens. We provide in Supplementary Information the Strehl ratio of the metalens for different values of $\sigma _D$ (SI, Fig. 4). These results confirm that a fabrication precision better than 10 nm is absolutely required to avoid a major degradation of the optical performance of the metalens.

## Conclusion

In this work, a dielectric metasurface based on silicon nanoresonators acting as a beam deflector in the near-infrared range has been designed, fabricated and characterized. The diffraction and deflection efficiencies are 94 % and 88 % respectively. The wavelength of maximum efficiency of the metasurface appeared to be slightly shifted spectrally with respect to the design wavelength. A systematic investigation of the influence of the different fabrication imperfections allows us to ascribe the observed spectral shift mainly to a residual slope of the lateral faces of the cylinders combined with a small fluctuation of the cylinder diameters. These results provide guidelines to design and fabricate metasurfaces implementing more complex optical functionalities and set the fabrication precision level that needs to be attained. In particular, the fabrication of optical metasurfaces operating in the visible to near-infrared range requires precisions on the sizes and edge slopes in the nanometer and degree range.

## Methods

### Finite difference time domain simulations

To compute the optical response of the metasurfaces, we use the Finite Difference Time Domain (FDTD) technique (open-source package MEEP^34^) throughout this study. The real part of the refractive index of polycrystalline silicon is taken from ellipsometry measurements performed on the layers used for the fabrication process. The imaginary part is taken from literature^39^. To compute the response of infinite square arrays of silicon nanocylinders of spacing *a*, we consider a unit cell of size $a\times a \times L$ with one cylinder at the center, perfectly matching layers in the (OZ) direction and periodic boundary conditions with a period *a* in the (OX) and (OY) directions. To compute the response of the metadeflector, we consider a unit cell of size $N_d a\times a \times L$ with $N_d$ cylinders at positions $x_i = ia$, perfectly matching layers in the (OZ) direction and periodic boundary conditions with a period $N_d \times a$ in the (OX) direction and *a* in the (OY) direction.

A plane wave with wavelength $\lambda _{c} = 750\ \hbox {nm}$ is incident normally on the metasurface and propagates towards positive *z*. The real and imaginary parts of the metasurface transmission coefficients, respectively noted $t_{r}$ and $t_{i}$, are extracted from the FDTD simulations. To do so, the electric field in an output plane $P_{out}$ parallel to the metasurface and located outside of the near-field region of the nanoresonators is first computed. The coefficients $t_{r}$ and $t_{i}$ are obtained by taking the ratio between the average electric field in the output plane $P_{out}$ with and without the metasurface. The Fresnel reflection by the first air/glass interface that would decrease the transmission efficiency by an additional 4 % is not taken into account. The transmission and phase-shift imprinted on the incident wave are then deduced as follows:

3 $$\begin{aligned} T= & {} |t_{r} |^2 + |t_{i} |^2 \end{aligned}$$

4 $$\begin{aligned} \Phi= & {} \arctan \left( \frac{t_{i}}{t_{r}} \right) \end{aligned}$$

## Supplementary Information

Supplementary Information.
