# Supplementary material for: Challenges in nanofabrication for efficient optical metasurfaces
Source: Sci Rep. 2021 Mar 10;11:5620. doi: 10.1038/s41598-021-84666-z (PMC7946922; doi:10.1038/s41598-021-84666-z)
Supplement: Supplementary file 1 — Supplementary Information. [file 41598_2021_84666_MOESM1_ESM.pdf]

Supplementary Information: Challenges in Nanofabrication for Efficient Optical Metasurfaces

Adelin Patoux[1,2,3], Gonzague Agez[1], Christian Girard[1], Vincent Paillard[1], Peter R. Wiecha[2], Aurélie Lecestre[2], Franck Carcenac[2], Guilhem Larrieu[2,4,*], and Arnaud Arbouet[1,*]

[1]CEMES-CNRS, Université de Toulouse, CNRS, Toulouse, France
[2]LAAS-CNRS, Université de Toulouse, CNRS, Toulouse, France
[3]Airbus Defence and Space, Toulouse
[4]LIMMS-CNRS/IIS, Institute of Industrial Science, The University of Tokyo, Tokyo, Japan
[*]arnaud.arbouet@cemes.fr, guilhem.larrieu@laas.fr

ABSTRACT

We provide additional information on the FDTD simulations and on the influence of the spacing between nanodisks on their optical properties. The influence of additional fabrication errors such as stitching during the electron beam lithography process and position of the nanocylinders are also discussed.

1 Influence of Nanocylinder Spacing on the Optical Properties

The spacing between nanocylinders influences the electromagnetic coupling between the nanoresonators and therefore their optical properties. Figure (1) shows the transmission efficiency and dephasing of an infinite square array of silicon nanocylinders with constant height H = 370 nm fabricated on a quartz substrate as a function of their spacing. The induced dephasing is relatively stable for lattice spacings larger than 250 nm. The transmission efficiency is more influenced with a minimum around 40% for lattice spacings around 400 nm. The spacing chosen in the main text (300 nm) yields the maximum transmission efficiency.

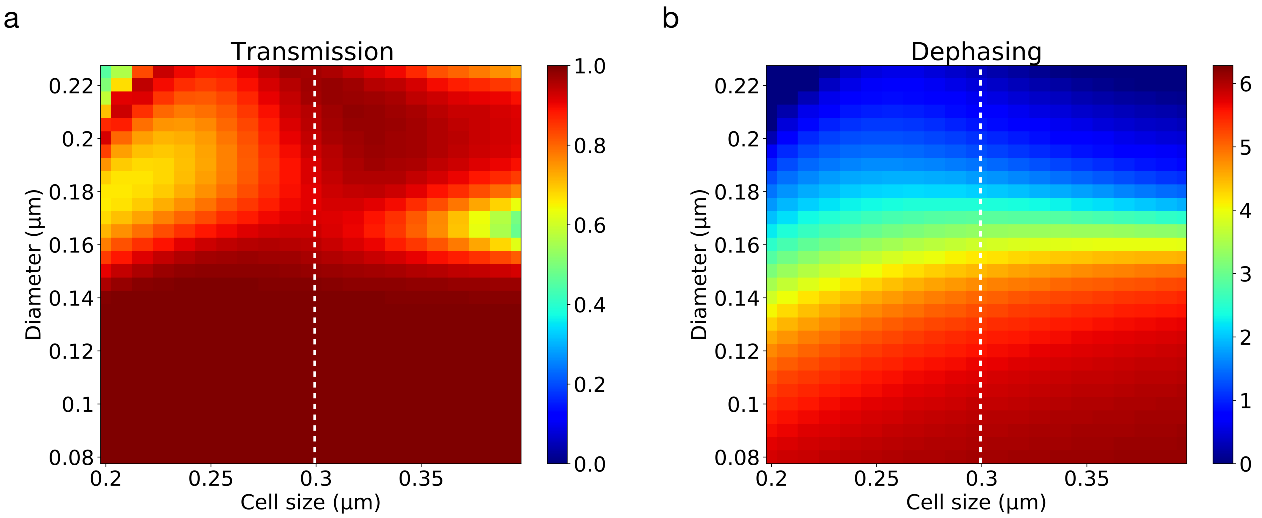

Figure 1. Transmission efficiency (a) and (b) dephasing of a $\lambda_c = 750$ nm plane wave normally incident on an infinite square array of silicon nanocylinders with constant height H = 370 nm.

2 FDTD simulations taking into account the combined influence of several fabrication errors

We have addressed in details the influence of the main fabrication errors in the main text. Our results show that the inclination of the side of the nanocylinders, combined with errors on their diameter and the presence of a silicon dioxyde layer has a significant influence on the optical response of the metasurface. To take into account the combined influence of these fabrication errors, we have computed the optical properties of the metadeflector taking as input the dimensions measured in the electron microscopy experiments. These FDTD simulations have been performed on an infinite square array with a supercell composed of 9 cylinders and an empty slot. The dimensions used in the simulations are indicated in the table (2-b) A 100 nm thick HSQ capping has been taken into account on top of the nanocylinders and an additional 10 nm thick silicon dioxyde layer was added on the cylinder sides. The height of the cylinders was $H = 390$ nm and the lattice spacing was 300 nm. The results are shown in Figure (2-c). They confirm the good agreement between the measured diffraction efficiency and the computed one. In particular, the observed spectral shift is well accounted for by these numerical simulations which take into account the geometry of the fabricated cylinders.

a

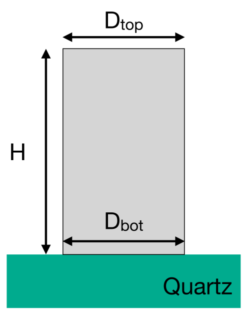

b

#	D_{bot}	D_{top}
1	210	224
2	183	194
3	180	185
4	175	183
5	157	167
6	156	160
7	140	150
8	138	146
9	107	121
10	Empty	Empty

c

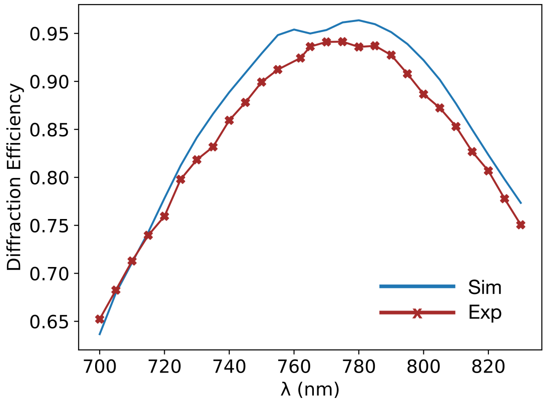

Figure 2. a) Conventions used for the FDTD simulations. b) Dimensions of the nanocylinders in the supercell as extracted from the electron microscopy experiments. c) Diffraction efficiency measured on the metadeflector as a function of the wavelength of the normally incident plane wave excitation (red line and symbols). Diffraction efficiency computed on an array of silicon nanocylinders illuminated by a normally incident plane wave as a function of wavelength (blue line). The simulated metasurface takes into account the dimensions measured in the TEM and the presence of a silicon dioxyde layer on the top and side of the nanocylinders.

3 Influence of Errors on the Nanodisk Position and Stiching Errors

We now consider the influence of errors on the position of the nanocylinders on the optical response of the metasurface. The metasurface is composed of a collection of silicon nanocylinders located at positions (x_i, y_i). The nano-objects are the sources of secondary waves with an amplitude and a dephasing governed by the size and spacing of the nanocylinders. Errors on the position of the nanocylinders can be accounted for by ascribing to the diffracting elements on the metasurface a modified value for their coordinates $(x'_i, y'_i) = (x_i + \delta x_i, y_i + \delta y_i)$. We have considered two different origins of errors. Random position errors are differences between the position of the fabricated nanocylinder with respect to its theoretical value. They vary randomly from one nano-object to the other with typical values smaller than 10 nm. They are typically a consequence of thermal, mechanical and electronic stability during the fabrication process. We have modelled the error on the nanodisk position by a change in position $(\delta^{pos} x_i, \delta^{pos} y_i)$ described by a gaussian distribution, the standard deviation σ_{pos} of which accounts for the precision of the fabrication process. The metasurface considered here has the same size ($100 \times 100 \mu$m^2), same supercell geometry as in the main text and operates at $\lambda_c = 750$ nm. The results shown in Figure (3-a) confirm that errors on the nanocylinder position have a weak influence on the metasurface response. A standard deviation as large as 40 nm on the nanocylinder position only decreases the deflection efficiency by 1 %.

Stitching errors are related to the electron beam lithography process. During this process, several regions are patterned sequentially, the sample table is mechanically moved between these regions. The transition from one writing field to another one can be accompanied by a shift of the coordinates with respect to the reference ones. An example of stitching error is given in Figure (3-b). To simulate these stitching errors, we splitted the entire metasurface of size $552 \times 552 \mu m^2$ in 8×8 different

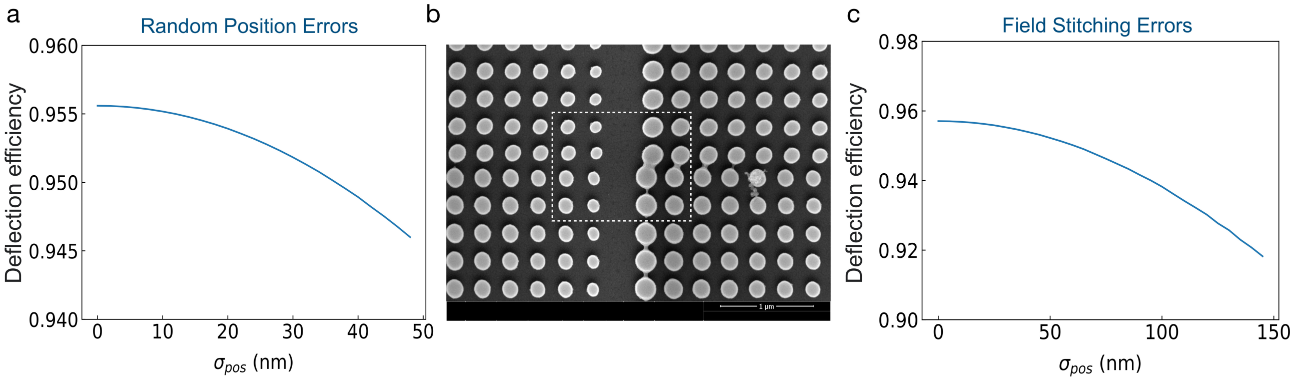

Figure 3. a) Deflection efficiency computed at $\lambda_c = 750$ nm as a function of σ_{pos}, the standard deviation on the nanocylinder position change $(\delta^{pos}x_i, \delta^{pos}y_i)$. The metadeflector has a size of $100 \times 100\mu\text{m}^2$ and has the same supercell has in the main text. b) SEM image of a deflector. An example of stitching error is visible in the framed region. c) Deflection efficiency as a function of σ_{pos}, the standard deviation on the shift of the writing field with respect to the expected value.

writing fields of size $69 \times 69\mu m^2$ to match our fabrication process. Each writting field is shifted to its reference position by $(\delta^{stit}x_I, \delta^{stit}y_I)$. Every nanocylinders in the same writting field are indentically shifted by $(\delta^{stit}x_I, \delta^{stit}y_I)$, we suppose here that there are no random position errors (discussed previously). To simulate stitching errors, we started with the simulation of a $69 \times 69\mu m^2$ deflector writing field the transmission of which can be written as $T_{\text{wf}}(u,v) = \iint t(x,y)e^{-2i\pi(ux+vy)}\text{d}x\text{d}y$.
The total response $T_{\text{tot}}(u,v)$ of the entire metasurface can then be obtained as:

$$T_{\text{tot}}(u,v) = T_{\text{wf}}(u,v)\sum_I e^{-2i\pi(ux'_I+vy'_I)} \tag{1}$$

Where $(x'_I, y'_I) = (x_I + \delta^{stit}x_I, y_I + \delta^{stit}y_I)$, and (x_I, y_I) are the reference positions for each writting field. The position errors are calculated according to a gaussian distribution of standard deviation σ_{pos} ranging from 0nm to 300nm. A total of 2000 simulations were done for each value of σ_{pos} to ensure validity of the results. The maximum values observed in our experiments are close to 70 nm (cf Figure (2-b)). As shown in Figure (2-c), these shifts only decrease the deflection efficiency by a few percents. The decrease in deflection efficiency observed in Figures (3-a-c) is due to the increasing disorder induced by larger values of σ_{pos} : part of the output beam energy is transferred from the diffracted beams to a diffusive background.

4 Optical Performance of a Metalens based on Silicon Nanocylinders: variation of the Strehl ratio with fabrication precision

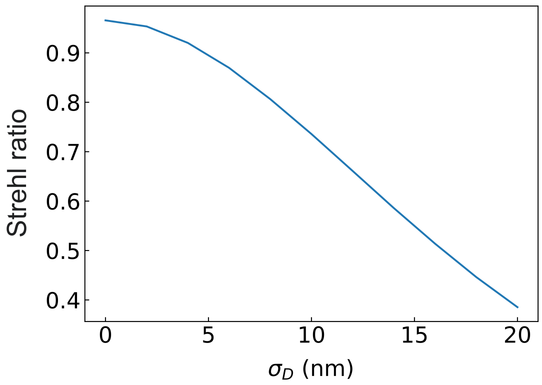

Figure 4. Variation of the Strehl ratio of a metalens with the standard deviation on the diameter of the nanocylinders.

The metalens has a diameter of 100μm and a focal distance of 500μm and is designed to operate at $\lambda_c = 750$ nm. It is composed of a collection of silicon nanocylinders with diameters chosen as explained in the main text. The focusing properties

of the metalens as a function of the errors on the nanocylinder diameter have been studied assuming that the diameters are distributed around their theoretical values according to a gaussian distribution of standard deviation σ_D. The Strehl ratio of the metalenses has been computed for different values of σ_D. The results are given in Figure 4 showing the rapid decrease of the performance of the metalens with the precision on the nanocylinder diameters.
